# Fabrication of TiC_x_-TiB_2_/Al Composites for Application as a Heat Sink

**DOI:** 10.3390/ma9080642

**Published:** 2016-07-29

**Authors:** Shili Shu, Hongyu Yang, Cunzhu Tong, Feng Qiu

**Affiliations:** 1 State Key Laboratory of Luminescence and Applications, Changchun Institute of Optics, Fine Mechanics and Physics, Chinese Academy of Sciences, Changchun 130033, China; shushili@ciomp.ac.cn (S.S.); tongcz@ciomp.ac.cn (C.T.); 2 Key Laboratory of Automobile Materials, Ministry of Education, and Department of Materials Science and Engineering, Jilin University, Changchun 130025, China; 3 Department of Mechanical Engineering, Oakland University, Rochester, MI 48309, USA; 4 Jiangsu Provincial Key Laboratory of Advanced Welding Technology, Jiangsu University of Science and Technology, Zhenjiang 212003, China

**Keywords:** composites, heat sink, wear, thermo-physics properties

## Abstract

Metal matrix composites reinforced with ceramic particles have become the most attractive material in the research and development of new materials for thermal management applications. In this work, 40–60 vol. % TiC_x_-TiB_2_/Al composites were successfully fabricated by the method of combustion synthesis and hot press consolidation in an Al-Ti-B_4_C system. The effect of the TiC_x_-TiB_2_ content on the microstructure and compression properties of the composites was investigated. Moreover, the abrasive wear behavior and thermo-physics properties of the TiC_x_-TiB_2_/Al composite were studied and compared with the TiC_x_/Al composite. The compression properties, abrasive wear behavior and thermo-physics properties of the TiC_x_-TiB_2_/Al composite are all better than those of the TiC_x_/Al composite, which confirms that the TiC_x_-TiB_2_/Al composite is more appropriate for application as a heat sink.

## 1. Introduction

Heat sinks are used to dissipate the heat generated from electronic components during their working process and protect them from external damage and mechanical forces [1,2]. Accordingly, ideal heat sink materials should exhibit superior thermal conductivity (TC) to effectively dissipate the heat generated by the electronic components and high strength and good abrasive wear behavior to protect the electronic components [3,4]. In addition, the heat sink materials also need possess a suitable coefficient of thermal expansion (CTE) to avoid high thermal stresses generated at the boundary between the heat sink and electronic components [5,6].

Aluminum possesses a relatively high TC and low density, which makes it become a material commonly used as heat sinks [7,8]. However, the CTE of aluminum (~23 × 10^−6^/°C) is so high compared to those of semiconductor materials (~7 × 10^−6^/°C) that thermal stress and strain may occur at the boundary between them [9,10]. Aluminum matrix composites offer the possibility of tailoring the properties of aluminum by adding an appropriate reinforcement phase to meet the demands for low CTE in thermal management [11]. Wei et al. [12] fabricated the Al-20Si matrix composites reinforced with SiC particles by high pressure solidification and investigated the effects of solidification pressure and SiC volume fraction on the thermal expansion behavior. They concluded that the CTE of the composite decreases with increasing the SiC content and the high pressure solidification technique would be helpful to develop the SiC/Al-Si matrix composite as a packaging material. Zhang et al. [13] investigated the effect of a metalloid silicon addition on the thermal-physical properties of diamond/Al composites. They reported that the silicon addition would effectively increase the TC and decrease the CTE of the diamond/Al composites. It can be seen from the above-mentioned work that the reinforcing particles used in aluminum matrix composites for heat sink applications are mainly limited to SiC and diamond, which are usually introduced into the Al matrix by an ex situ method. The shortcoming is that the interface bonding between the reinforcement and the Al matrix in the composites fabricated by the ex situ method is usually imperfect, which limits the further improvement of the mechanical and thermo-physical properties of the composites.

TiC_x_-TiB_2_ particles offer the advantages of enhanced strength, fracture toughness and wear resistance over the monolithic constituent (TiC_x_ or TiB_2_). In particular, the TiC_x_-TiB_2_ particle–reinforced metal matrix composites could be fabricated by an in situ method, which takes advantage of the cleaner particle-matrix interface and the better comprehensive properties. The TiC_x_-TiB_2_ particle-reinforced Ni, Fe and Mg matrix composites were extensively investigated as engineering materials [14,15,16]. However, to the authors’ knowledge, the research on the TiC_x_-TiB_2_ particle–reinforced aluminum matrix composites has mainly focused on the study of the microstructure and reaction mechanism [17,18]. Investigation on the properties and heat sink application of the TiC_x_-TiB_2_/Al composite has never been reported.

For heat sink materials, the aim of adding reinforcements is to decrease their coefficient of thermal expansion (CTE). A low content addition of reinforcement cannot decrease the CTE sufficiently. So, aluminum matrix composites with low content reinforcement (<40 vol. %) are usually used as engineering materials. Those reinforced with high content particles (>40 vol. %) are used as electric packaging materials. However, if the content of the reinforcements is too high (>60 vol. %), the thermal conductivity (TC) and processing ability of the composites become worse. So, in this paper, the content of the reinforcement was selected as 40–60 vol. %. The TiC_x_-TiB_2_/Al composites were fabricated by the in situ method of combustion synthesis combined with hot press consolidation in the Al-Ti-B_4_C system, and the effect of the TiC_x_-TiB_2_ content on the microstructure and compression properties of the composites was investigated. Moreover, the abrasive wear behavior and thermo-physics properties of the TiC_x_-TiB_2_/Al composite were studied and compared with the TiC_x_/Al composite. This will offer some guidance to fabrication and investigation for the aluminum matrix composite in a heat sink application, and even more to the other metal matrix composite in a heat sink application.

## 2. Experimental

### 2.1. Fabrication of the TiC_x_-TiB_2_/Al Composites

The raw materials used were commercial powders of aluminum (99% purity, ~47 μm), titanium (99.5% purity, ~25 μm) and B_4_C (98% purity, ~3.5 μm). The theoretical molar ratios of TiC_x_: TiB_2_ in the aluminum matrix composites were predetermined to be 1:2 with 40, 50 and 60 vol. % volume fraction based on the following reaction [19]:

*x*Al + 3Ti + B_4_C = TiC_x_ + 2TiB_2_ + *x*Al (1)

The blended powders of the Al-Ti-B_4_C systems were sufficiently mixed in a stainless steel die by ball milling for 8 h at a low speed (~35 rpm). After that, the mixtures were cold pressed into cylindrical compacts of 28 mm in diameter and 30 mm in height, and then the compacts were put in a self-made vacuum thermal explosion furnace. When the temperature which was measured by Ni-Cr/Ni-Si thermocouples suddenly rose rapidly, indicating that the formation reaction of the ceramics was ignited, the sample was quickly pressed just when it was still hot and soft, and then was cooled down to the ambient temperature. In addition, for comparison, the sample of 50 vol. % TiC_x_/Al composite was also fabricated by the above method in a Al-Ti-C system.

### 2.2. Characterization

The phase constituent of the products was investigated by X-ray diffraction (XRD, Moldel D/Max 2500PC, Rigaku, Tokyo, Japan) with Cu K_α_ radiation. The microstructure and worn abrasive surfaces were investigated by scanning electron microscopy (SEM, Moldel Evo18 Carl Zeiss, Oberkochen, Germany) and field emission scanning electron microscope (FESEM, JSM 6700F, JEOL, Tokyo, Japan).

### 2.3. Property Tests

Uniaxial compression tests were carried out under a servo-hydraulic materials testing system (MTS 810, MTS Systems Corporation, Minneapolis, MN, USA) with a strain rate of 1 × 10^−4^ s^−1^. Micro-hardness of the composites was measured by a Vickers hardness tester (Model 1600-5122VD, Newage, Feasterville, PA, USA) using a static load of 2 N and a dwell time of 15 s. The sizes of the sample for the compression and micro-hardness tests are 3 mm in diameter and 6 mm in a height.

Abrasive wear tests were conducted on a pin-on-disk machine under loads ranging from 5 to 25 N with the Al_2_O_3_ abrasive papers as counter face. The sizes of the sample for abrasive wear tests are 4 mm × 4 mm × 12 mm dimensions. The wear rate was defined as the volume loss suffered per unit sliding distance, and the volume loss is obtained from the ratio of weight loss to the density of the composites. The weight loss was measured using an electronic balance with a resolution of 0.1 mg, and the density (*ρ*) was measured by Archimedes’ water-immersion method.

The thermal conductivity, defined as K, can be calculated from K = *αρc* [20,21], where *α* is thermal diffusivity, *ρ* is density and *c* is specific heat. The thermal diffusivity was measured using a laser flash method (NETZSCH LFA427, NETZSCH, Selb, Germany), and the specific heat was determined using Differential Thermal Analysis (DTA, SDT-Q600, TA, New Castle, DE, USA) under argon atmosphere.

The coefficient of thermal expansions of the composites were measured between 100 °C and 300 °C using a Dilatometer (NETZSCH DIL402C, NETZSCH, Selb, Germany) at heating and cooling rates of 5 K/min under argon atmosphere. Each result presented here is an average of three distinct experiments. The sizes of the measured samples were 4 mm × 4 mm × 25 mm.

## 3. Results and Discussion

### 3.1. Phase Identification and Microstructures

**Figure 1:**
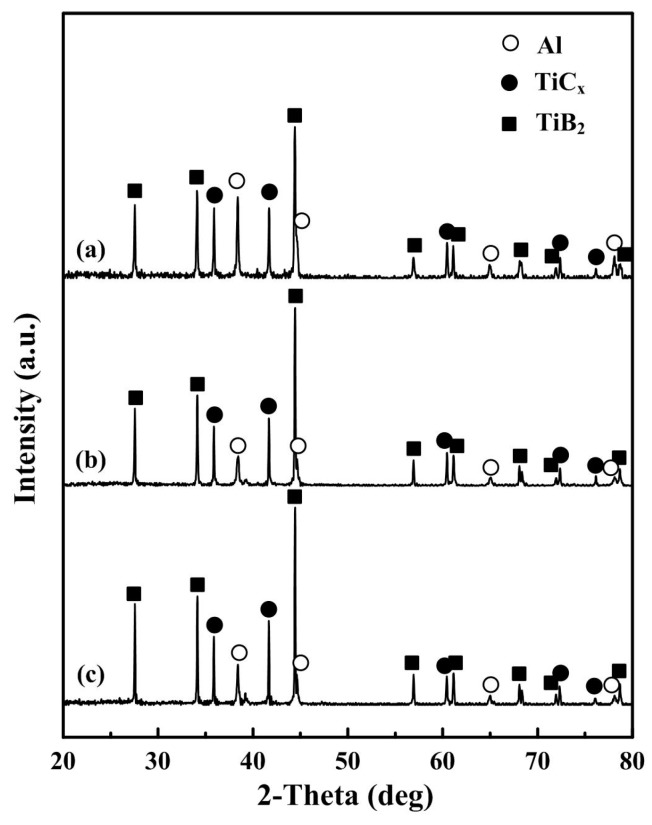
XRD patterns of (**a**) 40 vol. % TiC_x_-TiB_2_/Al; (**b**) 50 vol. % TiC_x_-TiB_2_/Al and (**c**) 60 vol. % TiC_x_-TiB_2_/Al composites.

Figure 1 shows the X-ray diffraction results for the composites reinforced with various TiC_x_-TiB_2_ contents. The products in these samples are mainly Al, TiC_x_ and TiB_2_ phases, without any intermediate phases that can be detected. It indicates that the pure in situ dual-reinforcement TiC_x_-TiB_2_/Al composites could be successfully fabricated by the method of combustion synthesis and hot press consolidation. In addition, as the Al content decreases, the content of the synthesized TiC_x_ and TiB_2_ would be increased consequently, which is consistent with the XRD results as shown in Figure 1 where the intensities of TiC_x_ and TiB_2_ peaks increase with the decrease in the Al content.

**Figure 2:**
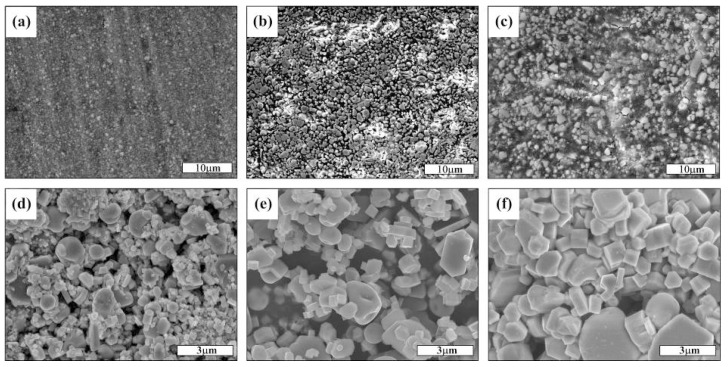
SEM images of the etched surfaces of (**a**) 40; (**b**) 50 and (**c**) 60 vol. % TiC_x_-TiB_2_/Al composites, and the FESEM images of the morphology of the extracted TiC_x_ and TiB_2_ particles from the (**a**) 40; (**b**) 50 and (**c**) 60 vol.% TiC_x_-TiB_2_/Al composites, respectively.

Figure 2a–c shows the SEM images of the etched surfaces of the TiC_x_-TiB_2_/Al composites with 40, 50 and 60 vol. % TiC_x_-TiB_2_ particles, respectively. As shown in Figure 2a–c, the composites have no appreciable porosity and the synthesized ceramic particles present a uniform distribution in the aluminum matrix. Figure 2d–f shows the FESEM images of the morphology of the extracted TiC_x_ and TiB_2_ particles from the composites with various ceramic contents, respectively. As indicated, the TiB_2_ particles formed in these composites are in the typical hexagonal prismatic or rectangular shape, while the TiC_x_ particles are in the spherical shape. As is known, the combustion temperature increases with the synthesized ceramic content [18,22], and the growth of the ceramic particles is an exponential function of the combustion temperature [23]; that is, the higher the combustion temperature is, the bigger the size of the ceramic particles will be. So, with the ceramic content increase from 40 to 60 vol. %, the size of TiB_2_ particles increases from 500 nm to 1 m, and the size of TiC_x_ particles increases from 1 μm to 3 μm.

### 3.2. Compression Properties

Besides heat dissipation, the other functions of the heat sink are used to protect electronic components from external damage and mechanical forces. Thus, heat sink materials also need to possess high strength and good abrasive wear behavior. Based on this consideration, the compression properties and abrasive wear behavior of the fabricated composites were studied in this section and the next section, respectively.

**Figure 3:**
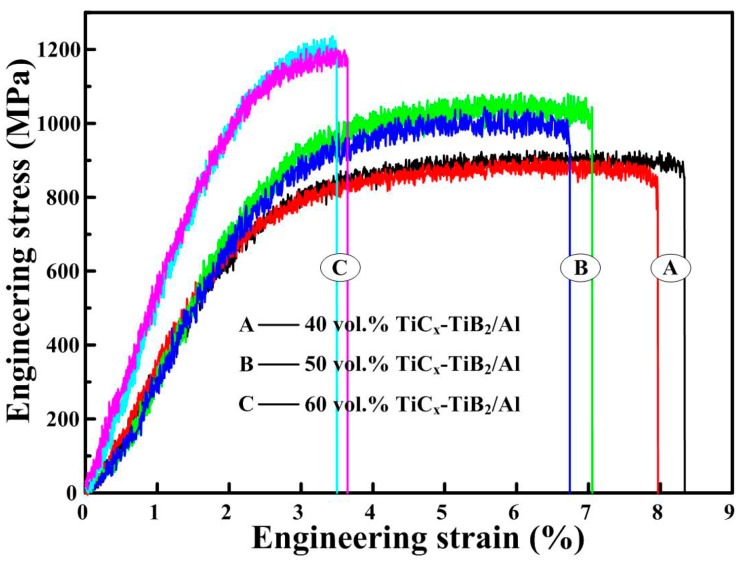
Compression engineering stress-strain curves of the TiC_x_-TiB_2_/Al composites.

Figure 3 shows the compression engineering stress-strain curves of the TiC_x_-TiB_2_/Al composites with various TiC_x_-TiB_2_ contents, and the compression properties and hardness of the composites are summarized in Table 1. The yielding strength (*σ*_0.2_), ultimate compression strength (*σ*_UCS_) and hardness (Hv) of the composites increase with the increase in the content of TiC_x_-TiB_2_ particles, while the fracture strain (*ε*_f_) decreases. Usually, the compression strength of the composites is mainly controlled by the content of the reinforcing particles. Thus, the *σ*_0.2_ and *σ*_UCS_ of the composites increase with the increase in the content of the TiC_x_-TiB_2_ particles.

**Figure 4:**
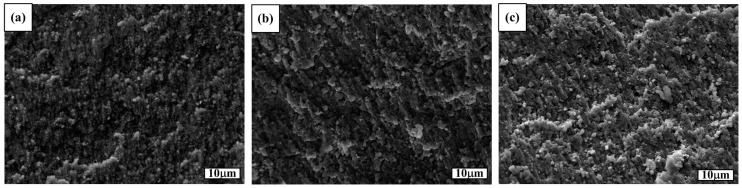
SEM images of the compression fracture surfaces of the (**a**) 40 vol. % TiC_x_-TiB_2_/Al; (**b**) 50 vol. % TiC_x_-TiB_2_/Al and (**c**) 60 vol. % TiC_x_-TiB_2_/Al composites.

Figure 4a–c shows the fracture surfaces of the TiC_x_-TiB_2_/Al composites. It can be seen from Figure 4a that all the TiC_x_-TiB_2_ particles are well enwrapped by the Al matrix, indicating that the split of the aluminum matrix is the main fracture mode for the 40 vol. % TiC_x_-TiB_2_/Al composite. However, it can be seen from Figure 4c that there are a lot of ceramic particles that exist on the fracture surface of the 60 vol. % TiC_x_-TiB_2_/Al composite. Thus, the debonding between the reinforcing particles and the Al matrix is the main fracture mode for the 60 vol. % TiC_x_-TiB_2_/Al composite. As shown in Figure 4b, both the above-mentioned fracture modes existed in the 50 vol. % TiC_x_-TiB_2_/Al composite. Thus, the Al content determines the ductility of the composites. With the increase in the TiC_x_-TiB_2_ content, the Al content reduces, leading to the decreasing fracture strain of the composites. Consequently, the 60 vol. % TiC_x_-TiB_2_/Al composite possesses the highest strength, but the worst ductility.

In general, the 50 vol. % TiC_x_-TiB_2_/Al composite exhibits better comprehensive compression properties. So, we thought the most suitable content of TiC_x_-TiB_2_ is 50 vol. % for the fabrication and application of TiC_x_-TiB_2_/Al composite. The yielding strength (*σ*_0.2_), ultimate compression strength (*σ*_UCS_) and hardness (Hv) of the 50 vol. % TiC_x_/Al composite fabricated in our previous work are 468 MPa, 714 MPa and 216.5 Hv, respectively [24]. The *σ*_0.2_, *σ*_UCS_ and hardness of the 50 vol. % TiC_x_-TiB_2_/Al composite are 315 MPa, 350 MPa and 81.3 Hv higher than those of the 50 vol. % TiC_x_/Al composite, respectively.

### 3.3. Abrasive Wear Properties

**Figure 5:**
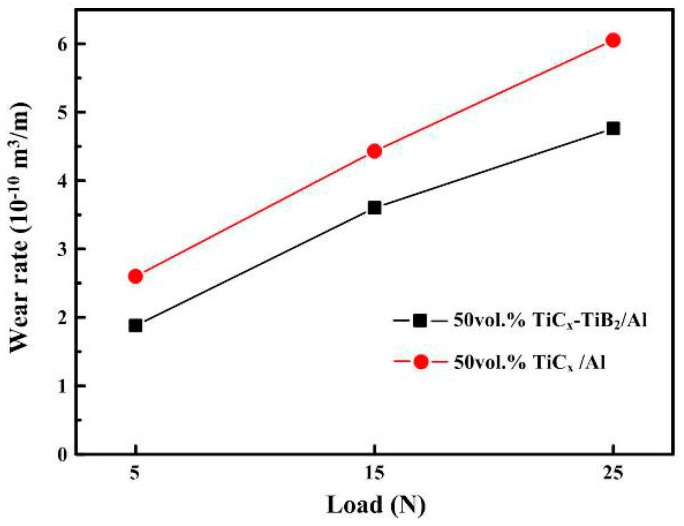
Wear rate vs. applied normal loads under the counter face of 10 m Al_2_O_3_ abrasive particles for the 50 vol. % TiC_x_/Al and 50 vol. % TiC_x_-TiB_2_/Al composites.

Figure 5 shows the variation in ware rate with the applied loads (5–25 N) for the 50 vol. % TiC_x_-TiB_2_/Al composite and the compared sample composite (50 vol. % TiC_x_/Al) tested against the Al_2_O_3_ abrasive particles of 10 μm. It is apparent that the wear rate of the two composites increases with the increase in the applied loads. The reason is that the penetration depth of the abrasive particles into the surface of the composites increases with the increase in the applied load, leading to the reinforcing particles being more easily spalled [25].

**Figure 6:**
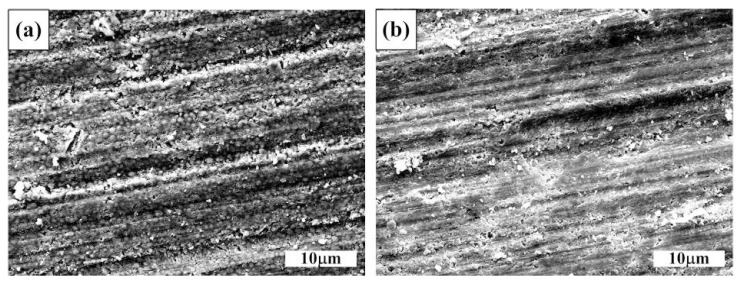
Worn surfaces of (**a**) 50 vol. % TiC_x_/Al and (**b**) 50 vol. % TiC_x_-TiB_2_/Al composites tested under the applied load of 15 N and the Al_2_O_3_ abrasive particle size of 10 m.

Another observation in Figure 5 is that the 50 vol. % TiC_x_/Al composite experiences an extremely high wear rate compared to the 50 vol. % TiC_x_-TiB_2_/Al composite under all applied loads. It indicates that the Al_2_O_3_ abrasives can penetrate easily into the 50 vol. % TiC_x_/Al composite during sliding, resulting in excessive material removal from the surface. This can also be confirmed by the worn surfaces of the composites which are shown in Figure 6a,b. As can be seen in Figure 6a, the Al_2_O_3_ particle has deeply penetrated into the 50 vol. % TiC_x_/Al composite, which could lead to the extensive deformation and fracture in the surfaces of the 50 vol. % TiC_x_/Al composite. The worn surfaces of the 50 vol. % TiC_x_-TiB_2_/Al composite shown in Figure 6b are relatively smoother than that of the 50 vol. % TiC_x_/Al composite. As discussed above, the hardness of the TiC_x_-TiB_2_/Al composite is higher than that of the TiC_x_/Al composite. Thus, the depth of the abrasive particles penetrating into the TiC_x_/Al composite would be deeper than that of the TiC_x_-TiB_2_/Al composite. According to these results, we presume that the reinforcing particles with different sizes and shapes possesses better properties effective in acting as a barrier to reduce the cutting efficiency of the Al_2_O_3_ abrasive particles and the plastic deformation of the aluminum matrix during wear. Thus, the abrasive wear resistance of the TiC_x_-TiB_2_/Al composite is better than that of the TiC/Al composite.

### 3.4. Thermo-Physical Properties

**Figure 7:**
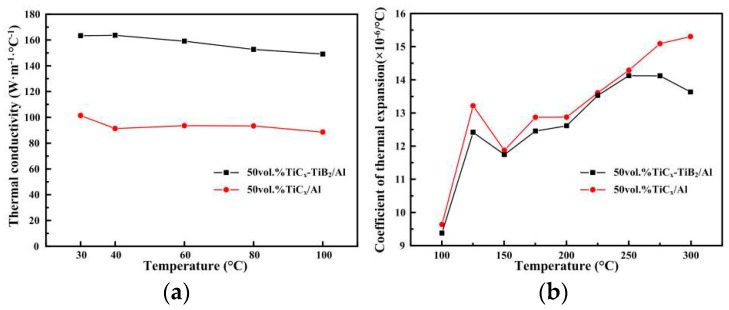
(**a**) Thermal conductivity vs. temperature and (**b**) coefficient of thermal expansion for the 50 vol. % TiC_x_/Al and 50 vol. % TiC_x_-TiB_2_/Al composites.

For heat sink materials, a high thermal conductivity (TC) and a low coefficient of thermal expansion (CTE) are often necessary for electronic packaging applications. The TC of TiC/Al and TiC_x_-TiB_2_/Al composite in the range of 30 °C to 100 °C is shown in Figure 7a. This temperature range was selected to fit the working temperature range for electronic packaging materials. The TC of the two composites decreases marginally with the increase in temperature up to 100 °C, and during the whole temperature range, the TC of the TiC_x_-TiB_2_/Al composite is higher than that of the TiC_x_/Al composite. The TiC_x_-TiB_2_/Al composite had a thermal conductivity of 160 W·m^−1^·°C^−1^ at the temperature of 30 °C, which, though lower than that of Al alloy (200 W·m^−1^·°C^−1^), is significantly higher than of the TiC_x_/Al composite (100 W·m^−1^·°C^−1^).

The CTE is expressed as the change in the dimensions of the material as a function of the temperature, which is desirable for electronic packing applications. In the present study, the coefficients of thermal expansion of the TiC_x_-TiB_2_/Al and TiC_x_/Al composites are 9.3 × 10^−6^/°C and 9.6 × 10^−6^/°C at the temperature of 100 °C, which is much smaller than that of Al alloy (~23 × 10^−6^/°C) [9]. This sufficient reduction of CTE indicates a perfect interface bonding between the particles and the matrix. Moreover, the CTE of the TiC_x_-TiB_2_/Al composite was about two-fifths of that of the Al alloy, which indicates that the TiC_x_-TiB_2_ particle filler effectively constrains the thermal expansion of the matrix. Moreover, during the whole range of 100 °C–300 °C, the CTE of the TiC_x_-TiB_2_/Al composite is totally lower than that of the TiC_x_/Al composite. This indicates that the dual-reinforcement TiC_x_-TiB_2_ particles are a promising filler to lower the CTE of electronic packaging materials.

Table 2 summarizes the thermo-physical properties of the aluminum matrix composites usually used in heat sink applications. It can be seen that the thermal conductivities of the fabricated aluminum matrix composites reinforced with different reinforcements are in the range of 120–321 W·m^−1^·°C^−1^ and the coefficients of thermal expansion are all larger than 10 × 10^−6^/°C. Thus, the TiC_x_-TiB_2_/Al composite fabricated in this work with the TC of about 160 W·m^−1^·°C^−1^ and CTE of 9.3 × 10^−6^/°C would satisfy the heat sink application requirements in electronic packaging.

## 4. Conclusions

The TiC_x_-TiB_2_/Al composites have been successfully fabricated by combustion synthesis and hot press consolidation in an Al-Ti-B_4_C system. The ceramic particles distribute uniformly in the composites and their sizes decrease with the increasing Al content. The compression strength and micro-hardness of the composites increase with the increasing TiC_x_-TiB_2_ content. The incorporation of TiC_x_ and TiB_2_ particles can act as a barrier to effectively protect the Al matrix from plastic deformation and destructive action, leading to the lower wear rate compared with the TiC_x_/Al composite. Moreover, the thermo-physical properties of the TiC_x_-TiB_2_/Al composites are more promising compared to the TiC_x_/Al composite. The combination of a TC of about 160 W·m^–1^·°C^–^^1^ and a CTE of 9.3 × 10^–6^/°C was achieved. The TE of the Al matrix would be restricted by the TiC_x_-TiB_2_ particles via the interfacial restriction effect. They are close to that of an Si chip and can meet the CTE requirements in electronic packaging well. The investigation demonstrated that the excellent mechanical and thermo-physical properties of TiC_x_-TiB_2_/Al composites make them ideal candidate materials for electronic packaging applications.

## Figures and Tables

**Table 1:** Room-temperature compression properties of the TiC_x_-TiB_2_/Al composites.

Sample	*σ*_0.2_ (MPa)	*σ*_UCS_ (MPa)	*ε*_f_ (%)	Hardness (Hv)
40 vol. %TiC_x_-TiB_2_/Al	657 ± 9	917 ± 7	8.1 ± 0.2	282.6
50 vol. %TiC_x_-TiB_2_/Al	783 ± 7	1064 ± 19	6.9 ± 0.2	297.8
60 vol. %TiC_x_-TiB_2_/Al	911 ± 4	1221 ± 14	3.6 ± 0.1	327.2

**Table 2:** Summary of the thermo-physical properties of the aluminum matrix composites usually used in heat sink applications.

Composites	Reinforcement Content	TC (W·m^−1^·°C^−1^)	CTE (10^−6^/°C)	Reference
AlN_p_/Al	50 vol. %	130	11.2	[26]
CNTs/Al	0.5–5 wt. %	120–199	-	[27]
SiC/Al	50–60 vol. %	165–224	-	[28]
	60 vol. %	190	10	[29]
	53–60 vol. %	151–216	-	[30]
diamond/Al	60 vol. %	130	17	[31]
	65 vol. %	210	13	[32]
	50 vol. %	321	13.2	[33]
